# Quantifying engineered nanomaterial toxicity: comparison of common cytotoxicity and gene expression measurements

**DOI:** 10.1186/s12951-017-0312-3

**Published:** 2017-11-09

**Authors:** Donald H. Atha, Amber Nagy, Andrea Steinbrück, Allison M. Dennis, Jennifer A. Hollingsworth, Varsha Dua, Rashi Iyer, Bryant C. Nelson

**Affiliations:** 1 Biosystems and Biomaterials Division, National Institute of Standards and Technology, Bld. 227, Rm. A247, MS 8313, 100 Bureau Drive, Gaithersburg, MD 20899 USA; 2 Bioscience Division, Los Alamos National Laboratory, Los Alamos, NM USA; 3 Center for Integrated Nanotechnologies, Materials Physics & Applications Division, Los Alamos National Laboratory, Los Alamos, NM USA; 4 Defense Systems and Analysis Division, Los Alamos National Laboratory, Los Alamos, NM USA; 5 Present Address: Navy Medical Research Unit–San Antonio, 3650 Chambers Pass, Bldg. 3610, Fort Sam Houston, TX 78234-6315 USA; 6 Present Address: Department of Biomedical Engineering and Division of Materials Science and Engineering, Boston University, Boston, MA USA

**Keywords:** Genotoxicity, Nanomaterials, Quantum dots, Cytotoxicity, Comet assay, Gene expression, Biomarkers

## Abstract

**Background:**

When evaluating the toxicity of engineered nanomaterials (ENMS) it is important to use multiple bioassays based on different mechanisms of action. In this regard we evaluated the use of gene expression and common cytotoxicity measurements using as test materials, two selected nanoparticles with known differences in toxicity, 5 nm mercaptoundecanoic acid (MUA)-capped InP and CdSe quantum dots (QDs). We tested the effects of these QDs at concentrations ranging from 0.5 to 160 µg/mL on cultured normal human bronchial epithelial (NHBE) cells using four common cytotoxicity assays: the dichlorofluorescein assay for reactive oxygen species (ROS), the lactate dehydrogenase assay for membrane viability (LDH), the mitochondrial dehydrogenase assay for mitochondrial function, and the Comet assay for DNA strand breaks.

**Results:**

The cytotoxicity assays showed similar trends when exposed to nanoparticles for 24 h at 80 µg/mL with a threefold increase in ROS with exposure to CdSe QDs compared to an insignificant change in ROS levels after exposure to InP QDs, a twofold increase in the LDH necrosis assay in NHBE cells with exposure to CdSe QDs compared to a 50% decrease for InP QDs, a 60% decrease in the mitochondrial function assay upon exposure to CdSe QDs compared to a minimal increase in the case of InP and significant DNA strand breaks after exposure to CdSe QDs compared to no significant DNA strand breaks with InP. High-throughput quantitative real-time polymerase chain reaction (qRT-PCR) data for cells exposed for 6 h at a concentration of 80 µg/mL were consistent with the cytotoxicity assays showing major differences in DNA damage, DNA repair and mitochondrial function gene regulatory responses to the CdSe and InP QDs. The BRCA2, CYP1A1, CYP1B1, CDK1, SFN and VEGFA genes were observed to be upregulated specifically from increased CdSe exposure and suggests their possible utility as biomarkers for toxicity.

**Conclusions:**

This study can serve as a model for comparing traditional cytotoxicity assays and gene expression measurements and to determine candidate biomarkers for assessing the biocompatibility of ENMs.

**Electronic supplementary material:**

The online version of this article (10.1186/s12951-017-0312-3) contains supplementary material, which is available to authorized users.

## Background

Engineered nanomaterials (ENMs) are widely used in commercial and industrial products in agriculture, engineering and medicine. The small size of ENMs provides them with special properties such as enhanced surface charge and a high surface area to volume ratio. Size and charge dependent interactions may increase the likelihood of biological effects on human cells [1]. Semiconductor nanocrystals, or quantum dots (QDs), are of particular interest in this regard due to their numerous applications in optics [2–4], biomedical diagnostics [5–7] and therapeutics [5, 8, 9]. This has created a critical need for the quantitative evaluation of ENM effects and determination of the sensitivity and reproducibility of the cytotoxicity assays used to measure these effects.

Many studies have focused on the toxicity of specific ENMs, using common cytotoxicity assays yet few detail the specific cellular mechanisms that play a role in their toxicity [10–13]. Gene expression analysis affords the opportunity to evaluate these mechanisms of toxicity, through the monitoring of regulatory genes that are affected. Cellular processes such as the induction of inflammatory cytokines, autophagy, necrosis, and apoptosis have been shown to be affected by physical properties of ENMS, like size and charge, as well as chemical properties, including the core composition and surface functionalization [12–16]. In this respect, it is critical to know how gene expression data can be correlated with common cytotoxicity assays, to know what genes will be useful to monitor as potential indicators of toxicity and to characterize the sensitivity and reproducibility of the measurements.

In the current study we compare four common cytotoxicity assays: the dichlorofluorescein assay for reactive oxygen species (ROS), the lactate dehydrogenase assay for membrane viability (LDH), the mitochondrial dehydrogenase assay for mitochondrial function, and the Comet assay for DNA stand breaks. We compared the responses of cultured normal human bronchial epithelial (NHBE) cells to two types of semiconductor QDs that were chosen based on their known difference in cytotoxicity: cadmium selenide (CdSe) QDs, which are known to produce significant toxic effects in cultured mammalian cells and indium phosphide (InP) QDs, which are reported to induce minimal toxicity to mammalian cells [10, 11, 17–22]. Considering these previous studies, CdSe and InP QDs functionalized with negatively charged mercaptoundecanoic acid (MUA) were chosen as well-characterized test materials to compare the results of the cytotoxicity assays and to determine if certain transcriptional changes related to DNA damage and repair and mitochondrial function can be used as predictive toxicological indicators in conjunction with prototypical cytotoxicity assays.

## Results

### Cytotoxicity measurements

All of the cytotoxicity and DNA damage data described in the following sections was collected using 5 nm diameter CdSe or InP cores rendered water soluble with MUA. MUA is a common thiol-based ligand used to stabilize colloidal QDs in aqueous media through the electrostatic repulsion of negative surface charge [23]. MUA itself was shown previously not to affect LDH release or DNA fragmentation [10]. The QD preparations tested here were characterized for UV absorbance, size and charge in aqueous media (see “Methods” section). A more detailed description of other experimental methods used here also can be found in “Methods” section.

**Fig. 1:**
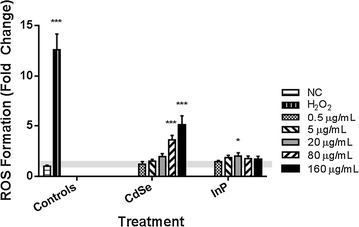
MUA-coated CdSe QDs cause increased ROS formation in NHBE cells. NHBE cells were incubated with increasing concentrations of 5 nm CdSe and InP QDs for 24 h. A significant concentration-dependent increase (p < 0.0001) in ROS formation was observed for cells treated with CdSe QDs compared to the medium only negative control (NC), shaded horizontal baseline and 100 μmol/L H_2_O_2_ positive control. InP QDs caused significant ROS after exposure to 20 μg/mL, but this response was not dose dependent. *p < 0.01, ***p < 0.0001. All experiments were independently repeated three times (n = 3). Error bars indicate one standard deviation from the mean (σ). Shaded baseline indicates expanded uncertainty (2σ)

NHBE cells were exposed to increasing concentrations (0.5–160 µg/mL) of either 5 nm CdSe or 5 nm InP QDs and quantitatively evaluated for reactive oxygen species (ROS) generation over 120 min. The ROS levels were determined using the fluorescent probe 5-(and-6)-chloromethyl-2′,7′-dichlorodihydrofluorescein diacetate (CM-H2DCFDA) measured at 10 min intervals. There was a significant increase in the ROS levels for the CdSe QDs for both the 80 and 160 µg/mL exposures (relative to media only controls), but minimal increases in the measured ROS levels for the InP QDs at these same exposure concentrations (Fig. 1).

**Fig. 2:**
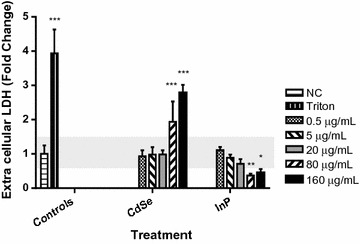
Concentration-dependent increase in extracellular lactate dehydrogenase (LDH) release for cells treated with CdSe QDsand lack of increase in LDH for cells exposed to increasing concentrations of InP QDs. NHBE cells were incubated with increasing concentrations of 5 nm QDs for 24 h. A significant concentration-dependent increase (p < 0.0001) in LDH release was observed for cells treated with CdSe QDs at 80 and 160 μg, without a corresponding increase for cells exposed to InP QDs, compared to the medium only negative control (NC), shaded horizontal baseline and 0.5% Triton-100 positive control. ***p < 0.0001; **p < 0.001; *p < 0.01. All experiments were independently repeated three times (n = 3). Error bars represent standard deviation from the mean (σ). Shaded baseline indicates expanded uncertainty (2σ)

NHBE cells were exposed to increasing concentrations (0.5–160 µg/mL) of either CdSe or InP QDs for 24 h prior to assessing cell necrosis using lactate dehydrogenase (LDH) activity as an indicator of cell membrane viability. A significant increase in LDH release with CdSe QD exposure (relative to media controls) was observed at QD concentrations greater than or equal to 80 µg/mL, without a corresponding increase with InP exposure, as indicated by asterisks (Fig. 2).

**Fig. 3:**
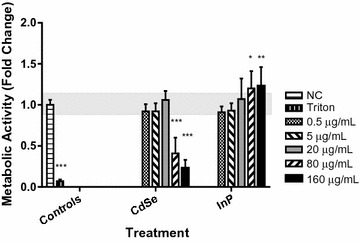
Dose-dependent decrease in mitochondrial function assay for cells treated with CdSe QDs and increase in mitochondial function for cells exposed to InP QDs. NHBE cells were incubated with increasing concentrations of QDs for 24 h. A significant dose-dependent decrease (p < 0.0001) in mitochondrial function was observed for cells treated with the highest concentrations of CdSe QDs, while a significant increase in mitochondrial function was noted for cells exposed to the highest concentrations of InP QDs, compared to the medium only negative control (NC), shaded horizontal baseline and 0.5% triton-100 positive control. ***p < 0.0001; **p < 0.001; *p < 0.01. All experiments were independently repeated three times (n = 3). Error bars represent standard deviation from the mean (σ), Shaded baseline indicates expanded uncertainty (2σ)

NHBE cells were exposed to increasing concentrations (0.5–160 µg/mL) of either CdSe or InP QDs for 24 h and then evaluated for cellular viability. Cellular metabolism was determined by measuring the conversion of the water-soluble tetrazolium dye (WST-1) to formazan by mitochondrial dehydrogenase enzymes. A 75% loss of function with CdSe exposure and a 25% increase in function with InP exposure (relative to media controls—shaded baseline) was observed at QD concentrations greater than or equal to 80 µg/mL, as indicated by asterisks (Fig. 3). The drop in metabolic function with CdSe at 80 µg or greater is consistent with the loss of cell viability, as evidenced by the increase in LDH release at these high concentrations of CdSe (Fig. 2). This effect has also been observed previously with CdSe-CYST [11].

### Measurements of DNA damage

**Fig. 4:**
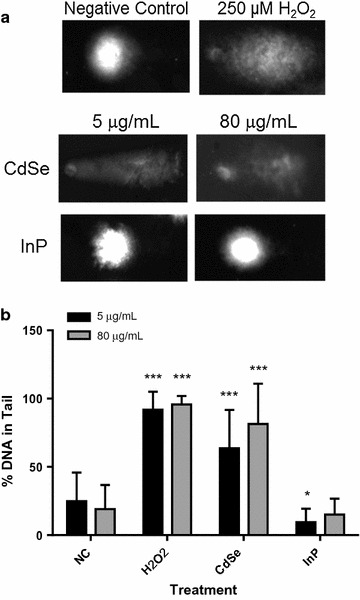
Comet assay of NHBE cells exposed to CdSe or InP QDs. NHBE cells were incubated with 5 or 80 µg/mL CdSe or InP QDs for 24 h and oxidative DNA damage (strand breaks) was measured by comet assay. **a** Typical microscopic images of single comets from cells exposed to CdSe or InP QDs compared to medium only negative and positive H_2_O_2_ controls. **b** A significant increase in (p < 0.0001) in DNA damage was observed for cells treated with both 5 and 80 μg/mL CdSe QDs compared to two sets of medium only negative controls (matched to 5 and 80 μg/mL experiments). Two sets of 250 μmol/L H_2_O_2_ positive controls (matched to 5 and 80 μg/mL experiments) are also shown for comparison. No DNA damage was apparent in cells exposed to InP QDs.***p < 0.0001; *p < 0.01. Error bars represent one standard deviation (n = 30 cells)

DNA damage (strand breaks) was measured by the alkaline Comet assay in NHBE cells exposed to CdSe and InP QDs. Cells were imaged by fluorescence microscopy after staining with SYBR Green I. Characteristic comet shapes after exposure resulting from increased mobility of the fragmented nuclear DNA was evident after exposure to the CdSe QDs (Fig. 4a). InP QDs showed minimal effect. Images are representative single cells of control groups (media only and H_2_O_2_-exposed cells) and QD-exposed cells. Data are expressed as percent of DNA in tail for QD-exposed NHBE populations compared to media only and H_2_O_2_ (250 µmol/L) controls. Significant differences between exposed cells and the media control are indicated by asterisks (Fig. 4b). DNA damage analyses revealed that CdSe QDs caused significant DNA strand breaks compared to InP QDs, which were equivalent to media-only treated cells. This response was observed in NHBE cells exposed to even the lowest concentration of CdSe QDs (5 µg/mL), which indicates the high sensitivity of the Comet assay.

### Effects on gene regulation

Cellular responses to a specific QD exposure can be considered both a function of QD concentration and the duration of the exposure. For our common direct cytotoxicity measurements, we chose a 24 h incubation at concentrations ranging from 0.5 to 160 µg/mL. In general, the QDs did not induce cytotoxic responses at (0.5, 5 or 20) µg/mL, therefore 5 µg/mL was selected as the low exposure test point in the gene expression study. For the high exposure test point, the cells showed increasingly reduced viability over 24 h when exposed to either 80 or 160 µg/mL CdSe QDs and the gene expression data could not be normalized using the actin gene. Hence, it was necessary to utilize a much lower exposure duration of 6 h and a high exposure concentration of 80 µg/mL in order to ensure suitable normalization of the gene expression data. The gene expression results should be considered an average of a mixed population of cells at various stages of response. Since the data are normalized using the actin gene, the cells contributing to the gene expression must have at least minimal machinery and ability to express the actin gene. However, many of the cells may have lost the ability (i.e., resulting from DNA damage) to express other genes. As in the direct cytotoxicity measurements, the gene expression analysis is used here as an indication of the average response of a mixed population of cells after specific QD exposure conditions.

For comparison to our direct cytotoxicity measurements, high-throughput quantitative reverse transcript polymerase chain reaction (qRT-PCR) was used to measure changes in expression of a selected panel of genes known to be involved in pathways of DNA damage and repair, mitochondrial function and proliferation. The gene expression changes were assessed with beta-actin as the normalizer reference gene using a 96.96 dynamic chip array as described previously [13]. NHBE cells were exposed to MUA-functionalized CdSe and InP QDs. To study the changes in gene expression, NHBE cells were exposed to a low (5 µg/mL), but persistent level of QDs for 24 h or a high (80 µg/mL) level of QDs for 6 h. At the low QD exposure level, genes related to DNA damage, DNA repair, mitochondrial function and proliferation (CDK1, Gadd45A, BRCA1, BRCA2, XPC, AHR, CYP1A1, CYP1B1, DHFR and VEGFA) were generally unchanged or down regulated relative to untreated controls for both QDs (Table 1). This is consistent with the LDH, ROS and mitochondrial cytotoxicity measurements in which significant cytotoxicity effects were only observed at QD concentrations above 5 μg/mL. When cells were exposed to the high (80 µg/mL) QD concentration, with the exception of DHFR, most of the genes associated with these pathways were significantly upregulated relative to untreated controls. Most of these genes were more upregulated with exposure to CdSe QDs than with InP QDs. The exception of genes XPC and UPC1 may be due to the loss of cell viability, metabolic function and in turn loss of gene expression in these pathways with exposure to high CdSe QDs. The cells exposed to the less toxic InP QDs appear to be capable of cellular responses in these genes, whereas the cells treated with the more toxic CdSe may not be capable of the same type of response. Other genes, such as SFN, BRCA2, CYP1B1, and VEGFA appear to be in stable cellular pathways that are activated upon CdSe treatment. GADD45A has been directly correlated to G2/M arrest under stress (excess Zn) in NHBE cells [24]. However, GADD45A gene expression was equivalent in both the CdSe and the InP QD exposed cells at 80 μg/mL, indicating that this pathway is unaffected.

**Table 1:** High-throughput qRT-PCR of NHBE cells treated with CdSe or InP QDs

Cell response pathway	Gene name	Fold change (5 µg/mL, 24 h)		Fold change (80 µg/mL, 6 h)	
		CdSe-MUA	InP-MUA	CdSe-MUA	InP-MUA
DNA damage	CDK1	*0.50* *±* *0.15*	*0.21* *±* *0.09*	1.92 ± 0.10	1.15 ± 0.01
	GADD45A	1.18 ± 0.02	1.00 ± 0.09	***4.17*** ***±*** ***0.95***	***4.95*** ***±*** ***0.66***
	SFN	1.50 ± 0.04	1.75 ± 0.04	***2.84*** ***±*** ***0.50***	0.86 ± 0.44
DNA repair	BRCA1	*0.55* *±* *0.07*	*0.18* *±* *0.03*	1.20 ± 0.05	0.96 ± 0.22
	BRCA2	*0.57* *±* *0.07*	*0.20* *±* *0.02*	***3.06*** ***±*** ***0.48***	*0.43* ± *0.17*
	XPC	0.89 ± 0.02	0.79 ± 0.05	0.84 ± 0.15	***2.29*** ***±*** ***0.15***
Mitochondrial function and repair	AHR	0.99 ± 0.04	1.11 ± 0.05	***2.18*** ***±*** ***0.29***	***2.35*** ***±*** ***0.43***
	CYP1A1	1.02 ± 0.11	1.17 ± 0.17	1.38 ± 0.35	*0.17* *±* *0.04*
	CYP1B1	1.29 ± 0.02	1.16 ± 0.07	***3.19*** ***±*** ***0.46***	0.70 ± 0.09
	DHFR	*0.57* *±* *0.02*	*0.40* *±* *0.02*	1.10 ± 0.10	1.15 ± 0.07
	UCP1	***2.29*** ***±*** ***0.50***	***2.20*** ***±*** ***1.64***	***4.32*** ***±*** ***0.87***	***7.20*** ***±*** ***0.51***
Proliferation	VEGFA	1.43 ± 0.05	1.44 ± 0.17	***2.18*** ***±*** ***0.48***	1.21 ± 0.16

Cells were incubated with 5 μg/mL CdSe or InP QDs for 24 h or with 80 μg/mL CdSe or InP QDs for 6 h. Gene expression changes for different classes of cellular functions and processes were measured by qRT-PCR. Values are expressed as fold change relative to media controls (= 1.0). Italics values indicates a twofold or greater decrease in gene expression relative to untreated cells (≤ 0.5) while bold italics highlights twofold or greater increases in gene expression relative to untreated cells (≥ 2.0). All experiments were independently repeated three times (n = 3). Errors represent one standard deviation

## Discussion

Compared to the CdSe QDs, InP QDs had minimal direct cytotoxic effects on the NHBE cells as measured by each of the common cytotoxicity assays. The increase in LDH release and ROS production that was observed with CdSe QD exposure was not observed upon exposure to InP QD. The correlation of LDH activity with the intracellular generation of ROS supports previous studies where QD cytotoxicity was found to be proportional to oxidative stress [25–27]. In addition, the minimal cytotoxic effects of the InP QDs also correlated with the DNA damage measurements showing minimal fragmentation/strand breaks with exposure to InP QDs. The common cytotoxicity measurements were also consistent in detecting the toxic effect of the CdSe QDs at the same range of concentration, and in a range consistent with earlier studies [10, 11].

Using CdSe QDs and InP QDs, we compared common cytotoxicity and gene expression measurements. The direct cytotoxic assays demonstrate that CdSe QDs induce severe DNA damage to NHBE cells when compared to InP QDs. The qRT-PCR gene expression data also revealed significant differences in certain DNA damage, DNA repair and mitochondrial function gene responses to CdSe and InP QDs, especially at higher concentration. The enhanced upregulation of markers BCR2, SFN, CYP1A1, CY1B1, CDK1 and VEGFA (Table 1) caused by CdSe QDs, indicate a consistent higher sensitivity and reaction to these QDs than for InP QDs. Based on our comparison to direct cytotoxicity measurements these genes, which are up-regulated specifically in response to increased CdSe QD exposure (i.e., BRCA2, SFN, CYP1A1, CYP1B1, CDK1 and VEGFA) may be possible biomarkers for cytotoxic damage from these types of ENMs.

Our study is a comparison of methods commonly used to determine NP toxicity instead of the determination of the individual cellular mechanisms of this toxicity. Although the gene expression data presented in this report yields useful information on the cellular responses to CdSe and InP QD exposure, the mechanisms of these responses remains unclear. For example, despite its lower toxicity, InP QDs induced a transcriptional response in NHBE cells for markers GADD45A and AHR equivalant to CdSe. Markers XPC and UCP1 were even more elevated in the case of InP QDs. One hypothesis is that the highly cytotoxic nature of CdSe QDs produces specific cellular damage that results in a reduced transcriptional response for certain markers such as XPC and UCP1. Cells exposed to the less toxic InP QDs, on the other hand, may be better able to respond in upregulating these markers. More extensive viability studies could be helpful to determine this. In addition, uptake studies would be helpful to determine the extent of internalization of the CdSe and InP NPs. Studies by Chau et al., using NHBE cells indicated it is the particle charge effects that affect the rate and route of transport [28]. However, the InP and CdSe nanoparticles used in the present study have the same MUA coating, which would be expected to have comparable properties of agglomeration and uptake. More extensive cellular response assays, such as time-dependent gene transcriptional profiles with additional markers, and alternative cell lines, could be performed on additional NPs at multiple concentrations to gain more insight into the mechanism of toxicity of ENMs.

## Conclusions

This study can serve as a model for the comparison of toxicology methods. In combination with traditional cytotoxicity assays, gene expression profiles can be used to determine candidate biomarkers which would be helpful in assessing the biocompatibility of ENMs. However, the use of gene expression measurements can yield results for certain genes which apparently are inconsistent with common cytotoxicity assays. Quantifying the cytotoxic interactions with cellular systems will require a thorough understanding of the biological responses produced by the ENMs.

## Methods

### QD preparation and characterization

#### QD synthesis

Cadmium oxide (CdO, 99.95%), and oleic acid (90%) were purchased from Alfa Aesar (Ward Hill, MA, USA), 1-octadecene (ODE, 90%), tetramethylammonium hydroxide (TMAH) and mercaptoundecanoic acid (MUA), from Acros Organics (Geel, Belgium), oleylamine (tech grade), selenium pellet (≥ 99.999%), myristic acid (≥ 98%), indium (III) acetate (99.99%), and dioctylamine (98%) from Aldrich (St. Louis, MO, USA), trioctylphosphine (TOP, 97%) trioctylphosphine oxide (TOPO, 90%), and tris(trimethylsilyl)phosphine ((TMS)_3_P; 98%) from Strem (Newburyport, MA, USA). All chemicals were used without any further purification.

CdSe (5 nm) QDs were synthesized as previously described [10]. Briefly, cadmium oleate was prepared by heating 1.45 g CdO in 20 mL oleic acid at 170 °C until colorless and cooled to 100 °C prior to degassing under a vacuum. TOP-Se was prepared in 50 mL TOP. 3.95 g Se pellets were dissolved in an inert atmosphere glovebox to make a TOP-Se solution. In an air-free environment, 1 g TOPO, 8 mL ODE, and 0.75 mL cadmium oleate were combined. The reaction mixture was thoroughly degassed at room temperature, and again at 80 °C. The temperature was increased to 300 °C under an atmosphere of ultra high purity argon. A solution of 4 mL of TOP-Se, 3 mL oleylamine, and 1 mL of ODE were combined and quickly injected into the cadmium oleate solution. The temperature was subsequently lowered to 270 °C for 1 min to control CdSe QD growth [29]. The solution was cooled, yielding CdSe QDs with a diameter of 5 nm. InP QDs were synthesized using a modification of an existing protocol [20]. A 0.08 mol/L solution of indium myristate (1:4.1 In:MA) was prepared by heating 2 mmol indium (III) acetate (584 mg), 8.2 mmol myristic acid (1.87 g), and 25 mL of ODE to 120 °C under vacuum. After 20 min, the solution was backfilled with argon and heated for another 2 h at 120 °C. In a 100 mL round-bottom flask, 5 mL of indium myristate was heated to 188 °C. A syringe containing 0.2 mmol (60 μL) of (TMS)_3_P and 1 mL di-*n*-octylamine was rapidly injected and the temperature stabilized at 178 °C. After one min, a second syringe containing 0.2 mmol (60 μL) (TMS)_3_P and 1 mL ODE were added dropwise at a rate of 1 mL/min. The reaction mixture was held at 178 °C for 15 min after the initial injection, when the heat was removed and the reaction was quenched with ~ 5 mL of degassed, room temperature ODE.

QDs were purified to remove excess ligands from the chemical synthesis as described [10]. QD concentrations were calculated according to Yu et al. [30] and Xie et al. [20] for CdSe and InP, respectively, on the basis of UV–VIS absorbance spectra. MUA was added to the toluene solution in amounts equivalent to 2 times the number of moles of QDs and incubated for 2 h. To facilitate the transfer of QDs from organic phase to water phase, a solution of TMAH in water (4 times the number of moles of QDs) was added dropwise. The water phase was precipitated with isopropanol, followed by centrifugation (5 min at 5000 rpm). The resulting pellet was redispersed in distilled water. Once the QDs transferred, the pH of the solution was brought back to ~ 6. Aggregates were carefully removed by centrifugation.

#### QD characterization

Absorption of aqueous suspensions of QDs were measured by UV–Vis spectroscopy and dynamic light scattering (DLS) using a Malvern Zetasizer [11]. Typical results in pure water are shown in Table 2 below.

**Table 2:** Characterization of InP MUA and CdSe MUA QDs

Label name	Total mass (g)	Total moles (mol)	Absorption max. (nm)	Size from UV–Vis (nm)	Size from DLS (nm)	Zeta potential (mv)
5 nm InP MUA	0.00197	6.778 × 10^−6^	613	4.61	3.99 ± 0.34	− 45.8 ± 8.7
5 nm CdSe MUA	0.0094	4.899 × 10^−5^	617	5.43	8.84 ± 2.45	− 54.9 ± 11.6

DLS measurements (mean and standard deviation) indicated minimal aggregation in pure water. Zeta potentials indicated high stability. However, extensive aggregation of MUA capped CdSe (622 ± 391 nm) was observed after 20 min in BEGM [11].

### Biological experiments

#### Cell culture and QD exposure

Normal human primary bronchial epithelial cells (NHBEs) were purchased from Lonza (Walkersville, MD, USA) and propagated in bronchial epithelial cell growth media (BEGM, Clonetics Bullet Kit Lonza, Walkersville, MD, USA) on 100 mm petri dishes coated with Type I 50 µg/mL rat tail collagen (BD Biosciences, Bedford, MA, USA) diluted in Dulbecco’s phosphate buffered saline (DPBS). Cells were passaged weekly and fed by replacing spent media with fresh media every (2–3) days. For necrosis, apoptosis, reactive oxygen species (ROS) production and mitochondrial function assays, cells from passages 3 to 7 were seeded at 2.5 × 10^4^ cells per well in 96-well flat bottom tissue culture plates and acclimated overnight. For comet assays and RNA isolation, cells were seeded at 1.5 × 10^5^ cells per well in 6-well tissue culture dishes. Cells were allowed to acclimate prior to QD exposures. QD suspensions ranging from 0.5 to 160 µg/mL and appropriate controls were prepared in DPBS or BEGM and immediately added to aspirated wells (150 µL/well for 96 well plates and 2 mL/well for 6-well plates). While the data is reported as µg/mL of QDs added to the cells, these concentrations equate to 0.3 to 97.0 µg/cm^2^ (96-well plates) and 0.1 to 33.3 µg/cm^2^ (6-well plates). Cells were incubated for 6 or 24 h in a humidified atmosphere at 37 °C and 5% CO_2_ during QD exposures.

#### Oxidative stress (ROS levels)

Intracellular ROS formation in NHBE cells exposed to QDs was quantified using 5-(and-6)-carboxy-2′,7′-dichlorodihydrofluorescein diacetate, acetyl ester (CM-H_2_DCFDA, Molecular Probes, Eugene, OR, USA). NHBE cells exposed to Dulbecco’s phosphate buffered saline (DPBS) only served as negative and 100 µmol/L H_2_O_2_ served as positive controls. QD controls at the highest concentrations were included in wells without cells to determine if QDs induce spontaneous fluorescence of CM-H_2_DCFDA. Fluorescence was measured using an excitation wavelength of 490 nm and an emission wavelength of 535 nm every 10 min post exposure for 120 min. Readings beyond 120 min resulted in errant readings due to cell starvation. Experiments were performed in triplicate on three independent occasions. Representative data from the 60 min reading are presented.

#### Cell viability assays

Cell membrane integrity was measured by assaying lactate dehydrogenase (LDH) activity in cellular supernatants. LDH kits were purchased from Roche (Indianapolis, IN, USA) [31, 32]. The 96-well plates were centrifuged at 200×*g*_n_ for 5 min to pellet uninternalized QDs. Supernatants (75 µL) were transferred to a clean plate, and LDH activity was assessed per the manufacturer’s instructions. Cells exposed to 0.5% Triton-100 were utilized as the positive control. Experiments were performed in triplicate on three independent occasions. QDs incubated with LDH reaction mix in a cell free environment were used to determine if QDs caused assay interference. Reactions were read colormetrically on a BioTek plate reader at an absorbance–wavelength of 490 nm and a reference wavelength of 600 nm after 15 min.

Mitochondrial activity, as measured using water-soluble tetrazolium dye (WST-1, Roche, Indianapolis, IN, USA), was assessed after incubation with QDs as described previously [10]. Cells exposed to 0.5% Triton-100 were utilized as the positive control. Experiments were performed in triplicate on three independent occasions. WST-1 reagent was added to each well (7.5 µL) of 96-well plates; plates were briefly vortexed and then incubated at 37 °C and 5% CO_2_ for 2–3 h prior to reading at an absorbance–wavelength of 420 nm and a reference wavelength of 600 nm. QD suspensions were also incubated with the WST-1 reagent alone to determine potential assay interference.

#### DNA damage

NHBE cells were exposed to QDs for 24 h, washed three times with DPBS, harvested by typsinization, counted and resuspended at 2.5 × 10^5^ cells/mL in freezing media consisting of 70% BEGM, 20% fetal bovine serum and 10% dimethyl-sulfoxide (DMSO) prior to storage in liquid nitrogen until comet assay analyses. Cells treated with media only or exposed to 250 µmol/L H_2_O_2_ for 1 h served as controls for DNA strand breaks. DNA strand breaks were measured by alkaline comet assay, otherwise known as single cell gel electrophoresis (SCGE) as described previously [11]. The percentage of DNA in the tail was calculated for each cell and averaged (n = 30 cells) for each treatment group. Percent DNA damage was determined as a function of treatment concentration and graphed as percent DNA in tail.

#### RNA isolation, high-throughput quantitative real-time polymerase chain reaction

NHBE cells were exposed to 5 or 80 µg/mL MUA InP or CdSe-QDs for 24 or 6 h, respectively. The number of viable cells was too low after treatment beyond 6 h at high QD concentrations. Cells were washed 3 times with DPBS to remove residual QDs prior to lysis. RNA was harvested and purified using Qiagen RNeasy mini-prep kits (Valencia, CA, USA) per manufacturer’s recommendations. For RNA samples used for transcriptomics, two DNA digestions were performed using Qiagen’s RNase free DNase set (Valencia, CA, USA). Gene expression changes for 96 targets were assessed using the BioMark real-time PCR high throughput chip system and 96.96 dynamic arrays (Fluidigm, CA, USA) as described previously [11]. The 96 TaqMan assays tested in this report include regulatory genes for pathways including mitochondrial function, inflammation, DNA damage and repair, autophagy and matrix formation. Real-time PCR was performed on the BioMark instrument using BioMark HD Data Collection Software v3.0.2. Data analyses were performed using Fluidigm Real Time PCR Analysis Software. Sample delta Ct values were calculated by using media only values as the negative control. Delta Ct values were calculated for the TaqMan assays using beta-actin as the normalizer reference gene.

RNA sequence experiments revealed many genes with altered expression. To select significantly regulated genes with confidence, we defined a gene as significantly regulated if it had an adjusted p value (p-adj) less than 0.05 (n = 3). This adjusted p value helps to lower the false positives, and is considered a more stringent test compared to the traditional p value [11]. Using p-adj with a threshold value of 0.05, we got a list of 118 genes of significant regulation. While not discounting the relevance of genes that do not show notable changes relative to media controls, we felt that focusing on the genes that were altered at least twofold would be more relevant. These 31 genes were found to be altered at least twofold at the high or low NP concentrations **(**see Additional file 1: Table S1). We then selected genes that we felt were the most relevant to compare with our cytotoxicity measurements. DNA damage and repair genes were selected for comparison to the comet assay. Mitochondrial function and metabolism genes were selected for comparison to our metabolic activity measurements and the proliferation gene was selected to compare with the extracellular LDH as an indicator of cell membrane viability.

### Statistical analyses

Biological data are presented as fold change above or below the media control and graphically represented as mean fold change. Statistical significance was calculated by One-way Analysis of Variance (ANOVA) using multiple comparisons versus control group (Bonferroni t-test). Analyses were performed using SigmaPlot version 11.0 (Systat Software, Inc., San Jose, CA, USA) using a minimum of three independent experiments for cell viability assays (LDH, mitochondrial function and apoptosis). Numerical transformations of the data were performed as necessary to satisfy equivalence of variance and normality parameters before statistical analyses were conducted. For the comet assays, statistical differences among treatment groups were evaluated by Student’s t test. p < 0.001 are indicated.

## Additional file

**Additional file 1: Table S1.** Quantitative RT-PCR of NHBE cells treated with CdSe or InP QDs cells.

## Abbreviations

QDs: quantum dots
ROS: reactive oxygen species
NHBE: normal human bronchial epithelial cells
DNA: deoxyribonucleic acid
MPA: mercaptopropionic acid
MUA: mercaptoundecanoic acid
CYST: cysteamine
53BP1: p53 binding protein 1
BEGM: bronchial epithelial cell growth media
LDH: lactate dehydrogenase
PBS: phosphate buffered saline
CAM: camptothecin
CM-H_2_DCFDA: 5-(and-6)-chloromethyl-2′,7′-dichlorodihydrofluorescein diacetate
WST-1: water-soluble tetrazolium
