# Supplementary material for: Quantifying engineered nanomaterial toxicity: comparison of common cytotoxicity and gene expression measurements
Source: J Nanobiotechnology. 2017 Nov 9;15:79. doi: 10.1186/s12951-017-0312-3 (PMC5679359; doi:10.1186/s12951-017-0312-3)
Supplement: Supplementary file 1 — Additional file 1: Table S1. Quantitative RT-PCR of NHBE cells treated with CdSe or InP QDs cells. [file 12951_2017_312_MOESM1_ESM.pdf]

Additional File 1

Quantifying Engineered Nanomaterial Toxicity: Comparison of Common Cytotoxicity and Gene Expression Measurements

Donald H. Atha, Amber Nagy, Andrea Steinbruck, Allison M. Dennis, Jennifer A. Hollingsworth, Varsha Dua, Rashi Iyer and Bryant C. Nelson

Table S1. Quantitative RT-PCR of NHBE cells treated with CdSe or InP QDs. Cells were incubated with 5 µg/mL CdSe or InP QDs for 24 h or with 80 µg/mL CdSe or InP QDs for 6 h. Gene expression changes for different classes of cellular functions and processes were measured by quantitative qRT-PCR. Values are expressed as fold change relative to media controls (=1.0). Blue text indicates a 2-fold or greater decrease in gene expression relative to untreated cells (<u><</u>0.5) while red text highlights two-fold or greater increase in gene expression relative to untreated cells (<u>></u>2.0). All experiments were independently repeated three times (n=3). Errors represent one standard deviation.

CELL RESPONSE PATHWAY	GENE NAME	FOLD CHANGE (5 µg/mL, 24 hrs)	
		5 nm CdSe-MUA	*5 nm InP-MUA*
**Adhesion and Invasion**	ITGA2	1.45 ± 0.13	3.01 ± 0.26
	MMP1	1.03 ± 0.05	3.26 ± 0.33
**Apoptosis**	BAK1	1.22 ± 0.51	1.11 ± 0.68
	BCL2	0.77 ± 0.04	0.47 ± 0.03
**Autophagy**	ULK1	1.28 ± 0.02	1.68 ± 0.06
	WIPI1	1.47 ± 0.03	1.82 ± 0.15
**DNA Damage**	CDK1	0.50 ± 0.15	0.21 ± 0.09
	GADD45A	1.18 ± 0.02	1.00 ± 0.09
	SFN	1.50 ± 0.04	1.75 ± 0.04
**DNA Repair**	BRCA1	0.55 ± 0.07	0.18 ± 0.03
	BRCA2	0.57 ± 0.07	0.20 ± 0.02
	XPC	0.89 ± 0.02	0.79 ± 0.05
**Mitochondrial Function and Metabolism**	AHR	0.99 ± 0.04	1.11 ± 0.05
	CYP1A1	1.02 ± 0.11	1.17 ± 0.17
	CYP1B1	1.29 ± 0.02	1.16 ± 0.07
	DHFR	0.57 ± 0.02	0.40 ± 0.02
	UCP1	2.29 ± 0.50	2.20 ± 1.64
**Proinflammatory Cytokines and Chemokines**	CSF2	1.50 ± 0.04	5.01 ± 0.35
	FAS	1.18 ± 0.06	1.06 ± 0.11
	IFNA1	2.02 ± 0.38	4.05 ± 0.44
	IL1A	1.43 ± 0.11	2.88 ± 0.03
	IL1B	1.11 ± 0.07	1.24 ± 0.06
	IL6	1.40 ± 0.07	1.02 ± 0.05
	IL8	1.27 ± 0.07	6.56 ± 0.34
	TNF	0.64 ± 0.10	1.18 ± 0.38
	CXCL1	1.27 ± 0.06	1.92 ± 0.15
**Proliferation**	VEGFA	1.43 ± 0.05	1.44 ± 0.17
**Signal Transduction and Transcription Factors**	FOS	0.91 ± 0.02	1.07 ± 0.06
	JUN	1.12 ± 0.17	1.26 ± 0.30
	MYC	0.96 ± 0.03	1.01 ± 0.04
	PIK3R1	0.93 ± 0.04	0.99 ± 0.09

CELL RESPONSE PATHWAY	GENE NAME	FOLD CHANGE (80 µg/mL, 6 hrs)	
		5 nm CdSe-MUA	*5 nm InP-MUA*
**Adhesion and Invasion**	ITGA2	1.18 ± 0.20	1.19 ± 0.22
	MMP1	1.33 ± 0.07	1.05 ± 0.11
**Apoptosis**	BAK1	0.59 ± 0.25	2.56 ± 0.40
	BCL2	0.63 ± 0.12	5.77 ± 1.43
**Autophagy**	ULK1	1.90 ± 0.35	2.93 ± 0.26
	WIPI1	1.21 ± 0.16	2.97 ± 0.47
**DNA Damage**	CDK1	1.92 ± 0.10	1.15 ± 0.01
	GADD45A	4.17 ± 0.95	4.95 ± 0.66
	SFN	2.84 ± 0.50	0.86 ± 0.44
**DNA Repair**	BRCA1	1.20 ± 0.05	0.96 ± 0.22
	BRCA2	3.06 ± 0.48	0.43 ± 0.17
	XPC	0.84 ± 0.15	2.29 ± 0.15
**Mitochondrial Function and Metabolism**	AHR	2.18 ± 0.29	2.35 ± 0.43
	CYP1A1	1.38 ± 0.35	0.17 ± 0.04
	CYP1B1	3.19 ± 0.46	0.70 ± 0.09
	DHFR	1.10 ± 0.10	1.15 ± 0.07
	UCP1	4.32 ± 0.87	7.20 ± 0.51
**Proinflammatory Cytokines and Chemokines**	CSF2	3.24 ± 0.59	0.19 ± 0.09
	FAS	0.67 ± 0.12	0.50 ± 0.04
	IFNA1	2.25 ± 0.22	1.79 ± 0.37
	IL1A	2.62 ± 1.16	1.76 ± 1.42
	IL1B	0.41 ± 0.09	0.37 ± 0.06
	IL6	0.82 ± 0.17	0.40 ± 0.12
	IL8	18.36 ± 3.79	2.53 ± 0.23
	TNF	6.50 ± 1.61	5.24 ± 0.74
	CXCL1	0.31 ± 0.07	0.27 ± 0.09
**Proliferation**	VEGFA	2.18 ± 0.48	1.21 ± 0.16
**Signal Transduction and Transcription Factors**	FOS	0.17 ± 0.03	0.52 ± 0.07
	JUN	2.24 ± 0.58	0.82 ± 0.21
	MYC	0.08 ± 0.01	0.28 ± 0.06
	PIK3R1	0.36 ± 0.05	0.52 ± 0.05
